# Synthesis of Carbon Foam from Waste Artificial Marble Powder and Carboxymethyl Cellulose via Electron Beam Irradiation and Its Characterization

**DOI:** 10.3390/ma11040469

**Published:** 2018-03-22

**Authors:** Hong Gun Kim, Yong Sun Kim, Lee Ku Kwac, Su-Hyeong Chae, Hye Kyoung Shin

**Affiliations:** 1 Institute of Carbon Technology, Jeonju University, 303 Cheonjam-ro, Wansan-gu, Jeonju-si, Jeollabuk-do 55069, Korea; hkim@jj.ac.kr (H.G.K.); wva223g6@naver.com (Y.S.K.); kwac29@jj.ac.kr (L.K.K.); 2 Department of BIN Convergence Technology, Chonbuk National University, 567 Baekje-daero, Deokjin-gu, Jeonju-si, Jeollabuk-do 54896, Korea; suc_0819@naver.com

**Keywords:** carbon foam, carboxymethyl cellulose, waste artificial marble powder, electron beam irradiation

## Abstract

Carbon foams were prepared by carbonization of carboxymethyl cellulose (CMC)/waste artificial marble powder (WAMP) composites obtained via electron beam irradiation (EBI); these composites were prepared by mixing eco-friendly CMC with WAMP as the fillers for improved their poor mechanical strength. Gel fractions of the CMC/WAMP composites obtained at various EBI doses were investigated, and it was found that the CMC/WAMP composites obtained at an EBI dose of 80 kGy showed the highest gel fraction (95%); hence, the composite prepared at this dose was selected for preparing the carbon foam. The thermogravimetric analysis of the CMC/WAMP composites obtained at 80 kGy; showed that the addition of WAMP increased the thermal stability and carbon residues of the CMC/WAMP composites at 900 °C. SEM images showed that the cell walls of the CMC/WAMP carbon foams were thicker more than those of the CMC carbon foam. In addition, energy dispersive X-ray spectroscopy showed that the CMC/WAMP carbon foams contained small amounts of aluminum, derived from WAMP. The results confirmed that the increased WAMP content and hence increased aluminum content improved the thermal conductivity of the composites and their corresponding carbon foams. Moreover, the addition of WAMP increased the compressive strength of CMC/WAMP composites and hence the strength of their corresponding carbon foams. In conclusion, this synthesis method is encouraging, as it produces carbon foams of pore structure with good mechanical properties and thermal conductivity.

## 1. Introduction

Carbon foams are lightweight materials containing highly porous spherical voids and a three dimensional web construction with a large external surface area, low thermal expansion coefficient, and tunable thermal conductivity [1,2,3]. These properties enable the use of carbon foams for numerous applications such as energy storage, filtration, aeronautics, and environmental protection catalysis [4,5,6,7,8]. However, the application as the medium and insulating materials in the construction of carbon foams can be limited because of their poor mechanical strength and high manufacturing costs and during manufacturing [9,10,11]. Carbon foams are usually prepared using coal, pitch, and thermosetting plastics derived from fossil fuels as precursors [12,13,14,15]. However, fossil fuel-derived precursors produce toxic gases during combustion; therefore, it is necessary to find new precursor materials derived from biopolymers to reduce the emission of toxic gases, and fillers to also improve the mechanical strength.

Carboxymethyl cellulose (CMC) as precursor of carbon foam is an eco-friendly material and a representative cellulose derivative. It is a white, non-toxic, water-soluble, light-weight, and thermally stable powder and has been used in the food, agricultural, pharmaceutical, and medicine industries [16,17].

Waste artificial marble powder (WAMP) as filler to improve the mechanical property is a byproduct formed during the shaping of artificial marble. Artificial marble is cheaper and lighter and exhibits a better mechanical performance than natural marble; therefore, it is widely used in the construction and kitchen industry. Moreover, as the production of artificial marble increases, the amount of WAMP produced during cutting and lapping also increases. Generally, large amounts of WAMP are generally not reused, and then are disposed or incinerated in landfill sites, resulting in air and soil pollution as well as excessive waste [18,19,20]. Because of these serious environmental consequences, some researchers are studying its availability than disposal or incineration. Therefore, effective and eco-friendly reuse and recycle of WAMP is necessary. Electron beam irradiation (EBI) can be used for crosslinking composites. EBI facilitates easy polymerization and cross-linking without the need for environmental and temperature control, and has many advantages such as a short manufacturing time, energy efficiency, a simple process [21,22,23].

In this study, CMC/WAMP carbon foam with three-dimensional reticular structure can be applied as reinforced matrix of the construction with fire-resistance, sound absorption and insulation, and so on. To improve the poor mechanical properties of CMC carbon foam which can limit its application and development, we used WAMP as the filler to enhance the mechanical properties of carbon foams. We prepared carbon foams through the carbonization of CMC/WAMP composites obtained using EBI. By varying the various EBI doses and calculating the gel fraction, the CMC/WAMP composites with the highest gel fraction value was carbonized at 1000 °C. Further, the obtained carbon foams were characterized by thermogravimetric analysis (TGA), scanning electron microscopy (SEM), energy dispersive X-ray spectroscopy (EDS), thermal conductivity, and compressive strength testing.

## 2. Experimental

### 2.1. Materials

CMC with 1000–2000 (cps) of viscosity at 40 °C was purchased from DAEJUNG Co. WAMP was supplied by the LION CHEMTECH Corporation, Korea. Citric acid as cross-linking agent was of analytical grade and was used as received.

### 2.2. Preparation of CMC Composite, CMC/WAMP Composites and Their Carbon Foams

For obtaining CMC composite and CMC/WAMP composites, 10 wt % CMC and 4 wt % citric acid were dissolved in distilled water at room temperature and 0, 1, 2, and 3 wt % WAMP were added to the above pastes and the resulting pastes was stirred until WAMP was uniformly dispersed. The obtained CMC/WAMP pastes were irradiated at doses of 20, 40, 60, 80, and 100 kGy at room temperature under atmospheric pressure. EBI was performed using a conveyor-type scanned beam with an accelerating voltage of 1.14 MeV of, a beam current of 7.6 mA, and a dose rate of 6.67 kGy/s. EBI-treated CMC and CMC/WAMP pastes were then freeze-dried to obtain CMC composite and CMC/WAMP composites. These composites were carbonized for 60 min at a high temperature of 1000 °C under pure nitrogen (99.999%) in a tubular furnace to produce CMC/WAMP carbon foams.

### 2.3. Analysis

To measure the degree of cross-linking in the CMC/WAMP composites, gel fractions in distilled water were investigated, and the soluble parts were extracted after treatment for 24 h at room temperature. The residual gel fractions were completely dried at 80 °C under vacuum. The gel fraction values were obtained by the equation

Gel fraction (%) = (insoluble parts/weight of the sample) × 100

TGA (TG/DTA STA 6000, Perkin-Elmer, Buckinghamshire, UK) was conducted at a heating rate of 10 °C/min. SEM/EDS images were obtained to observe the micro-porous structure, morphology, and elemental composition using a CX-200TA (Daejeon, Korea). Thermal conductivity was measured by TPS 2500, Hot Disk, Sweden. Compressive strength testing was conducted on an Instron 5567 tester (Instron, USA) according to the ASTM standard C365.

## 3. Results and Discussion

### 3.1. Gel Fraction of CMC/WAMP Composites Obtained at Different EBI Doses

**Figure 1:**
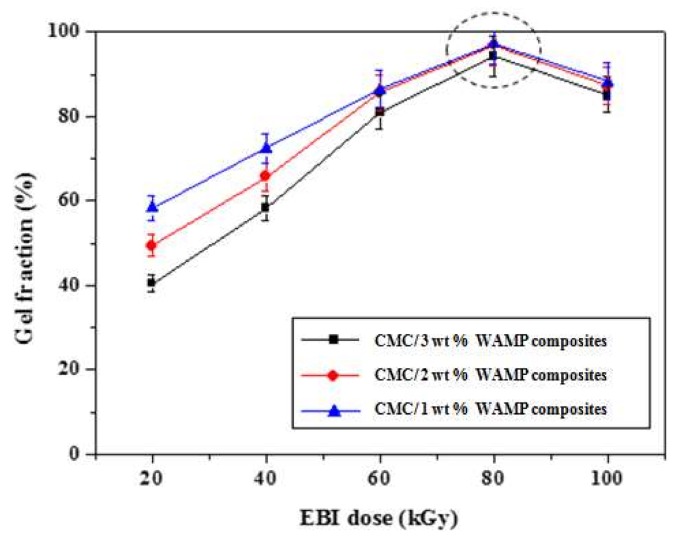
Effect of EBI dose on gel fraction of CMC/WAMP composites.

The gel fraction generally represents the transformation of a material from a linear structure to a cross-linked structure. Therefore, for the preparation of carbon foams resistant at temperatures higher than 1000 °C from CMC/WAMP composites, gel fraction becomes very important. Figure 1 presents the effect of EBI dose on the gel fraction of the CMC/WAMP composites. The gel fraction of of CMC/WAMP composites increased with increasing EBI doses to 80 kGy, after which the gel fraction decreased. This was because excessive EBI dose causes oxidation, leading to chain scission. Overall the maximum gel fraction achieved was over 95% at an 80 kGy; hence, these composites were selected for carbonization. CMC composite was also prepared at 80 kGy of same condition, and then was carbonized to compare with CMC/WAMP composites.

### 3.2. TGA Analysis of CMC/WAMP Composites

**Figure 2:**
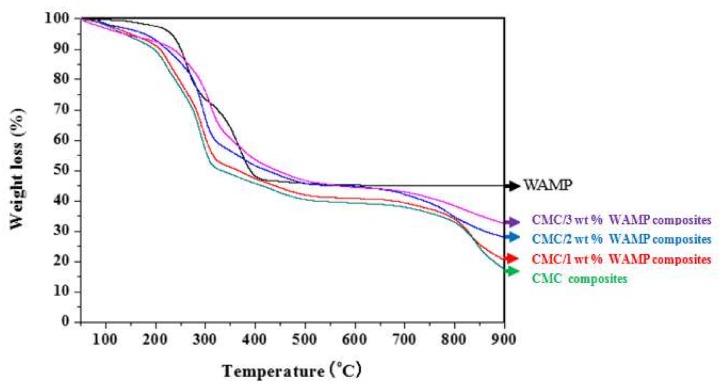
TGA curves of CMC/WAMP composites obtained at EBI dose of 80 kGy.

The thermal behavior for WAMP, CMC composite, and CMC/WAMP composites in terms of the weight changes in samples versus heating temperature is presented in Figure 2. WAMP represented a steep thermal decomposition behavior in the range of around 200–400 °C and a near-constant residue of around 45% from 400 to 900 °C. The CMC composite was found to lose weight due to water evaporation below 200 °C and thermal decomposition above 200 °C; thus, the residue at 900 °C was 17.4%. The thermal behavior of the CMC/WAMP composites was similar to that of the CMC composite. However, we could observe that the increase in the WAMP content increased the thermal stability and the residue amount observed at 900°. These results indicated that a stable and large residue (45.1%) of the WAMP component in the composites at temperature above 400–900 °C influenced the final residue of the CMC/WAMP composites.

### 3.3. SEM/EDS Analysis

**Figure 3:**
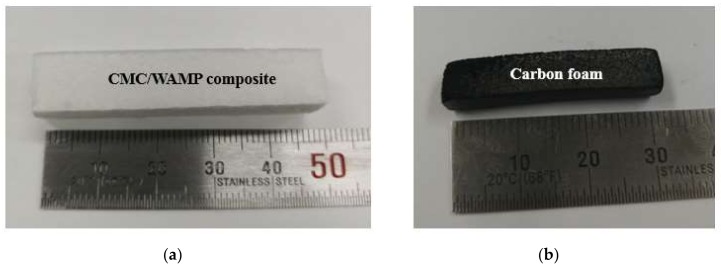

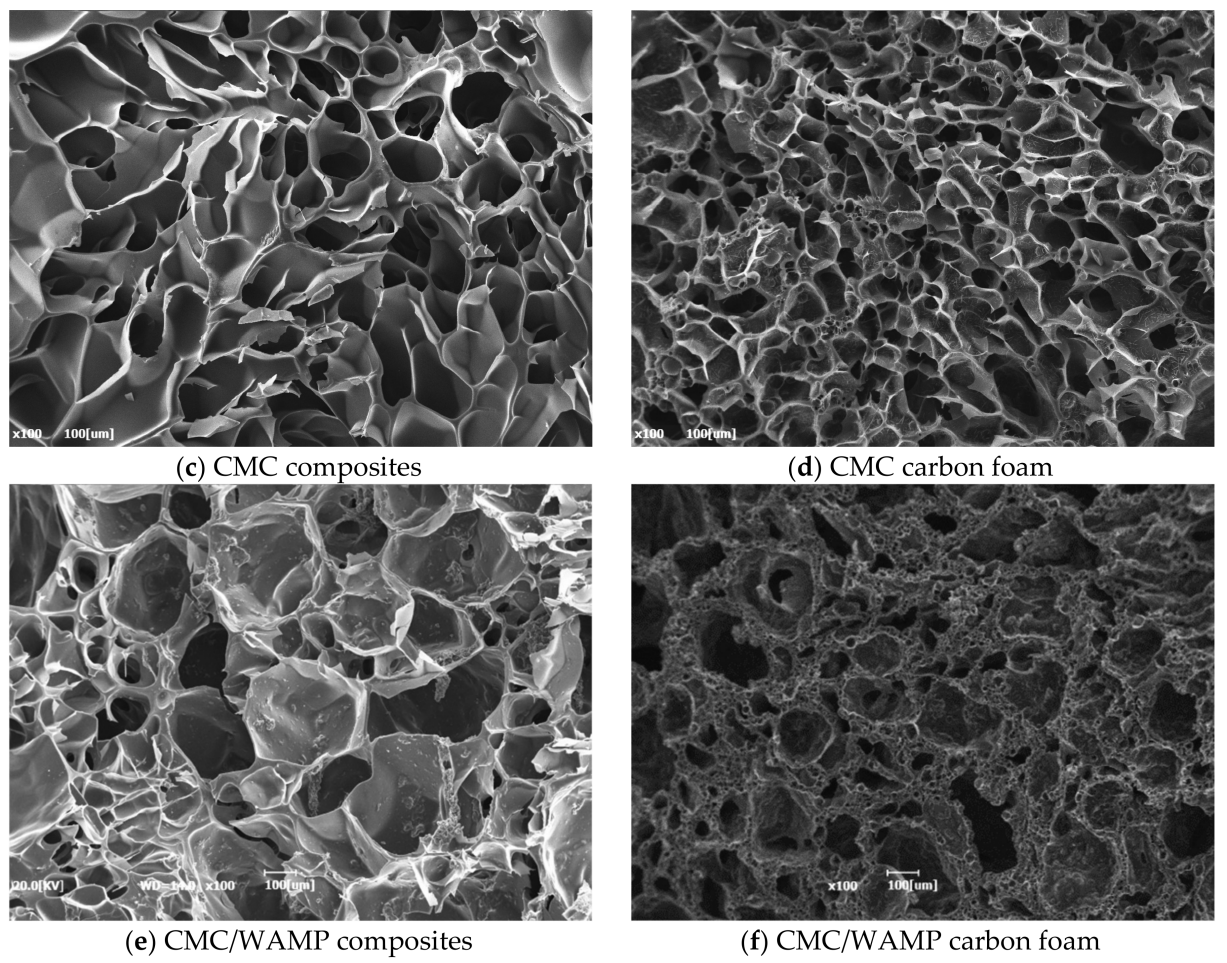
Photos (**a**,**b**) and SEM images (**c**–**f**) of CMC composite and CMC/WAMP composites and thus their carbon foams.

Figure 3 shows the SEM images of the cross-sectional morphologies of the composites and their carbon foams, Table 1 displays the elemental composition of carbon foams obtained by EDAX. From Figure 3, the CMC composite and CMC/WAMP composites had large and non-uniform pores with thin walls; the obtained carbon foams maintained this three dimensional structure without special defects, although the composites were carbonized at a high temperature of 1000 °C without stabilization. However, the pore sizes of the carbon foams decreased due to thermal shrinkage during carbonization and their densities increased. In particular, the cell walls of the CMC/WAMP carbon foams were thicker than those of the CMC carbon foam. From Table 1, we found that the CMC/WAMP carbon foams contained small amounts of aluminum. As the amount of WAMP increased, the aluminum increased, which in turn affected the thermal conductivity.

### 3.4. Thermal Conductivity

**Figure 4:**
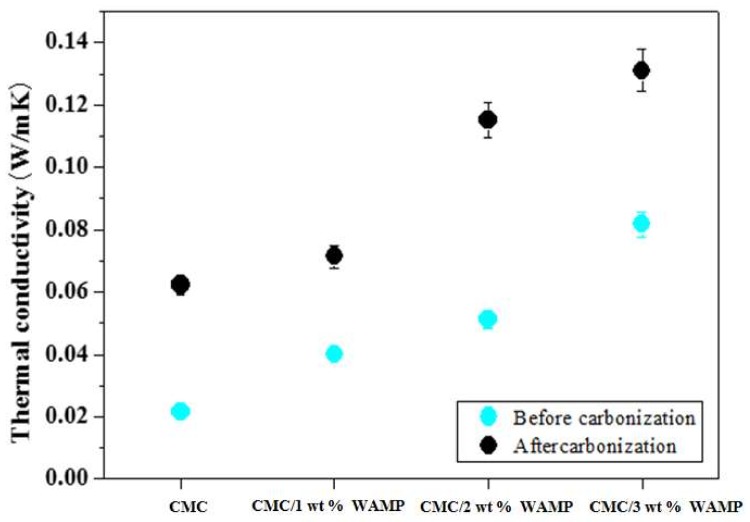
Thermal conductivity of CMC composite, CMC/WAMP composites, and their carbon foams.

Figure 4 shows the thermal conductivity of the CMC composite, the CMC/WAMP composites, and their carbon foams. Generally, the density, porosity, and fillers of the foam materials influence the thermal conductivity, and the presence of carbonized materials increase the thermal conductivity. The thermal conductivities of carbon foams were higher than those of the composites. In addition, thermal conductivities of the CMC/WAMP composites increased with increasing WAMP content, and the same trend was observed in their corresponding carbon foams. As mentioned in Table 1, WAMP contained aluminum, which contributed to the thermal conductivity of the CMC/WAMP carbon foams.

### 3.5. Compressive Strength

**Figure 5:**
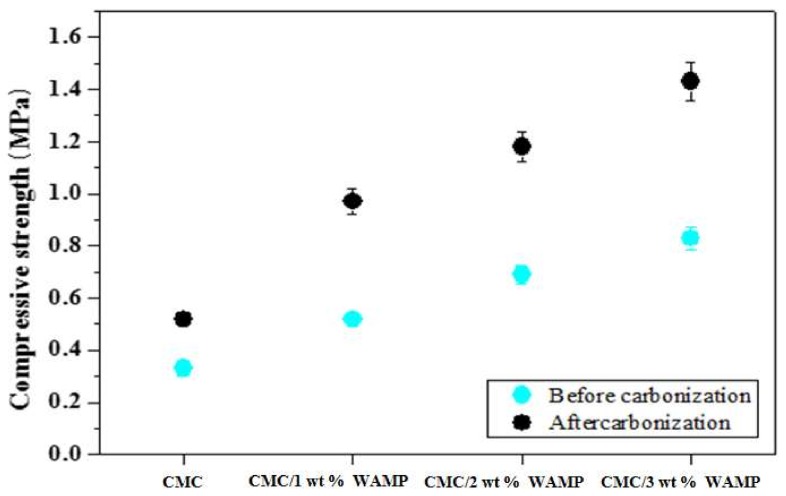
Compressive strength of CMC composite, CMC/WAMP composites, and their carbon foams.

Figure 5 represents the Compressive strength of the CMC composite, CMC/WAMP composites, and their carbon foams. As shown in Figure 5, the compressive strength of the composites was below 1 MPa but the increase in the WAMP content increased the compressive strength. In addition, the compressive strength of the carbon foams was about two times greater than those of their corresponding composites. Usually, compressive strength can be improved by the addition of fillers to endure the compressive load. Therefore, the compressive strength increased with increasing WAMP content. However, compared to carbon foams (over 2 MPa) prepared by fossil fuel precursor with several filler, the compressive strength values of CMC/WAMP carbon foam were very low [24,25,26].

## 4. Conclusions

Carbon foams were prepared by the carbonization of CMC/WAMP composites obtained via EBI of mixing CMC with WAMP. This experiment proposed a facile and economical method to synthesize carbon foams with high mechanical strength and using eco-friendly CMC as the precursor and WAMP as the fillers. The gel fractions of the CMC/WAMP composites increased with increasing EBI doses up to 80 kGy and subsequently decreased because of chain scission caused by excessive EBI. The maximum gel fraction was obtained at 80 kGy and was over 95%; therefore, composites obtained at an EBI dose of 80 kGy were carbonized. TGA analysis of these CMC/WAMP showed that the increase in WAMP content increased the thermal stability and the amount of residue at 900 °C. The SEM images showed that the cell walls of the CMC/WAMP carbon foams were thicker than those of the CMC carbon foam. In addition, EDS showed that the CMC/WAMP carbon foams contained small amounts of aluminum content increased with the WAMP content and resulted in increased thermal conductivities. The addition of WAMP also increased the compressive strength of the CMC/WAMP composites and that of the corresponding carbon foams. The results showed that this facile synthesis method by EBI uses CMC and effectively reuses and recycles WAMP to produce carbon foams with good mechanical properties and thermal conductivity although they showed small values compared to carbon foams prepared by fossil fuel precursor with several filler.

## Figures and Tables

**Table 1:** Elemental composition of carbon foams (weight, %).

Carbon Foam	Carbon (C)	Oxygen (O)	Sodium (Na)	Aluminium (Al)
CMC	50.01	30.62	19.37	0
CMC/1 wt % WAMP	31.51	38.12	25.81	4.57
CMC/2 wt % WAMP	41.71	35.40	17.30	5.60
CMC/3 wt % WAMP	52.48	27.04	12.58	7.90
